# Life Course Socioeconomic Status and Late-Life Cognition and Cognitive Decline in the Rush Memory and Aging Project

**DOI:** 10.1093/geroni/igaa057.831

**Published:** 2020-12-16

**Authors:** Anna Krasnova, Sarah Tom, Linda Valeri, Maria Glymour, Paul Crane, David Bennett

**Affiliations:** 1 Columbia University Mailman School of Public Health, New York, New York, United States; 2 Columbia University, New York, New York, United States; 3 University of California, San Francisco, California, United States; 4 University of Washington, Seattle, Washington, United States; 5 Rush University Medical Center, Chicago, Illinois, United States

## Abstract

The relationships among life course socioeconomic status (SES) measures with later life cognition and cognitive decline are unclear. We test the hypothesis that life-course SES is associated with late life level of cognition and rate of cognitive decline. The Rush Memory and Aging Project enrolled 1,864 dementia-free people aged ≥65 years between 1994 – 2018. Participants reported early life (parental education, number of siblings, and childhood financial need), mid-life (income at 40 years), and late life (baseline income) SES. Global cognitive function is a composite of 19 neuropsychological tests, administered annually. We utilized marginal structural models to assess the effect of SES (dichotomized at the median) at three life-course stages on late life global cognitive function and decline. We calculated inverse probability weights to adjust for socio-demographic confounders at each life-course stage. A total 1,063 participants had all relevant variables. Average follow-up was 4.4 years, and mean baseline age was 80.4 years. Most respondents were non-Hispanic white (89.7%) and female (74.1%). In the fully adjusted model, high childhood SES (coefficient 0.10; 95% CI 0.01, 0.20) and high late-life SES were associated with higher cognition intercept (coefficient 0.21; 95% CI 0.09, 0.32). High mid-life SES was associated with slower rate of cognitive decline (coefficient 0.02; 95% CI 0.001, 0.05). Childhood and late-life SES measures were not related to cognitive decline. Childhood and adult SES may reflect processes in building cognitive capacity, while midlife SES may reflect cognition maintenance. Interventions relating to SES across the life-course may benefit later life cognition.

